# Biofilm formation and transcriptome analysis of *Streptococcus gallolyticus* subsp. *gallolyticus* in response to lysozyme

**DOI:** 10.1371/journal.pone.0191705

**Published:** 2018-01-26

**Authors:** Imke Grimm, Jessika Dumke, Jens Dreier, Cornelius Knabbe, Tanja Vollmer

**Affiliations:** Institut für Laboratoriums- und Transfusionsmedizin, Herz- und Diabeteszentrum Nordrhein-Westfalen, Universitätsklinikum der Ruhr-Universität Bochum, Bad Oeynhausen, Germany; Oregon Health & Science University, UNITED STATES

## Abstract

*Streptococcus gallolyticus* subsp. *gallolyticus* is a commensal bacterium of the human gastrointestinal tract, and a pathogen causing infective endocarditis and other biofilm-associated infections via exposed collagen. This study focuses on the characterization of the biofilm formation and collagen adhesion of *S*. *gallolyticus* subsp. *gallolyticus* under different conditions. In this study, it has been observed that the isolate UCN 34 is resistant to 20 mg/ml lysozyme in BHI medium, whereas the strain BAA-2069 builds more biofilm in the presence of lysozyme compared to in a control of BHI without lysozyme. A transcriptome analysis with whole genome microarrays of these two isolates in BHI medium with lysozyme compared to control without lysozyme revealed changes in gene expression levels. In the isolate BAA-2069, 67 genes showed increased expression in the presence of lysozyme, while in the isolate UCN 34, 165 genes showed increased expression and 30 genes showed decreased expression through lysozyme treatment. Products of genes which were higher expressed are in involved in transcription and translation, in cell-wall modification, in hydrogen peroxide resistance and in bacterial immunity. Furthermore, the adhesion ability of different strains of *S*. *gallolyticus* subsp. *gallolyticus* to collagen type I and IV was analyzed. Thereby, we compared the adhesion of 46 human isolates with 23 isolates from animals. It was shown that the adhesion ability depends significantly on whether the isolate was isolated from human or animal. For example, high adhesion ability was observed for strain UCN 34 isolated from an infective endocarditis patient, whereas strain DSM 16831 isolated from koala feces adhered only marginally to collagen. Full genome microarray analysis of these two strains revealed strain-dependent gene expression due to adhesion. The expression of 25 genes of a transposon and 15 genes of a phage region in strain DSM 16831 were increased, which corresponds to horizontal gene transfer. Adherence to collagen in strain UCN 34 led to higher expression of 27 genes and lower expression of 31 genes. This was suggestive of a change in nutrient uptake.

## Introduction

Biofilm formation is a survival strategy for pathogens on non-biological surfaces (e.g., polystyrene) or in the host (e.g., extracellular matrix) [1]. It protects the bacteria from degradation through harsh environments, such as a low pH, hydrogen peroxide (H_2_O_2_), antibiotic treatment or other host defense mechanisms [2,3]. Biofilm formation starts with loose attachment of microorganisms to a surface, followed by durable adhesion to this surface and forming a community in an extracellular matrix [4].

Infective endocarditis (IE) is a biofilm-associated disease involving infection of the endocardial surface of the heart. Sterile inflammation of the endocardium or implantable cardioverter-defibrillator can be the initial factors of this disease [5]. Bacterial cells enter the bloodstream and can adhere to the altered endothelial surface, which consists of collagens, laminin, vitronectin and fibronectin [6]. Inflammatory processes stimulate the clotting process, which hides the colonizing bacteria with fibrinogen and platelets from immune cells in blood and tissue. Thereby, the vegetation develops with layers of bacteria and the thrombus [7]. Therefore, not only the extracellular matrix of the bacterial biofilm, but also host proteins and cells incidentally support the bacteria to survive within the host [8].

*Streptococcus gallolyticus* subsp. *gallolyticus* is a commensal bacterium of the human and animal gastrointestinal tract but it is also an opportunistic pathogen. It is the causative agent of IE in up to 10% of human cases [9]. IE caused by this bacterium is often associated with colon carcinoma [10–13]. Boleij et al. hypothesized that the bacterium translocates paracellularly by a carcinoma through the damaged epithelia of the colon, whereby the bacteria enter the blood circulation and are transported to the altered heart surface [14]. Consequently, the adherence to collagen at the damaged epithelium of the colon and the endocardium are important factors for the establishment of the disease [15]. It was also observed that *S*. *gallolyticus* subsp. *gallolyticus* causes other diseases associated with collagen exposure, such as spondylodiscitis, knee arthroplasty and endophthalmitis [16–19]. Vollmer et al. and Sillanpää et al. revealed that *S*. *gallolyticus* subsp. *gallolyticus* has more adhesion capability to collagen type I compared to other components of the extracellular matrix [20,21], whereas Sánchez-Díaz et al. detected higher adhesion to collagen type IV than type I [22]. Danne et al. showed that Pil1 (major pilin) expression is necessary for adherence to collagen type I [23]. Furthermore, *S*. *gallolyticus* subsp. *gallolyticus* forms biofilm on polystyrene [20,21].

Macrophages also play an important role at the site of an IE infection [24]. It was observed that macrophages, in addition to monocytes and neutrophilic granulocytes, are the most important producers of lysozyme in the immune system [25]. It was shown that microbial agents, like bacterial DNA or LPS, stimulate lysozyme release from these cells [26]. Lysozyme is an important enzyme for the immune system because it has a cationic microbial peptide activity and hydrolyses peptidoglycan, leads to cell death and lysis, and, therefore inhibits biofilm formation in, for example, *Staphylococcus aureus* [27,28]. Consequently, bacteria have developed different mechanisms to become resistant to lysozyme [29]. It was shown that the survival ability of *S*. *gallolyticus* subsp. *gallolyticus* in lysozyme-supplemented medium is strain-dependent [30].

This study focuses on the strain-dependent adhesion of *S*. *gallolyticus* subsp. *gallolyticus* to collagen. Additionally, the effect of lysozyme and hydrogen peroxide (H_2_O_2_) on *S*. *gallolyticus* subsp. *gallolyticus* biofilm formation on polystyrene was analyzed. We used full genome microarrays to find new aspects on lysozyme resistance and collagen adhesion of the IE pathogen *S*. *gallolyticus* subsp. g*allolyticus*. For most analyses, three isolates from human IE patients and two isolates from feces of koala or calf for comparison were used because the genomes of these five strains have been completely or partially sequenced [31–35].

## Material and methods

### Cell culture and bacterial strains

Strains of *S*. *gallolyticus* subsp. *gallolyticus* (Table A in S1 File) were grown in brain-heart infusion broth (BHI; Thermo Scientific, Waltham, USA) at 37°C and 220 rpm for overnight cultures. Bacterial cultures in the exponential growth phase were generated by inoculating 5 ml BHI medium with 100 μl overnight culture. The exponential growth phase was reached after 2.5 h at 37°C and 220 rpm. The bacterial titer was determined by serial dilutions in Dulbecco’s phosphate-buffered saline (DPBS) and plating 100 μl of an adequate concentration in triplicate on tryptone soya agar (Thermo Scientific, Waltham, USA). Tryptone soya agar plates were incubated at 37°C for at least 24 h and the colonies produced were counted using an aCOLyte colony counter (Synbiosis, Cambridge, UK).

### Adherence to collagen

96-well plates were coated with 0.1 mg/ml collagen type I, collagen IV from human placenta (Sigma, Steinheim, Germany) or 0.1 mg/ml bovine serum albumin (BSA) as a control in DPBS (Thermo Scientific, Waltham, USA) at 4°C overnight [20]. Solutions were discarded and non-specific binding sites were blocked for 2 h at 4°C with 250 μl blocking solution consisting of DPBS supplemented with 1% BSA and 0.05% Tween-20. The wells were then washed twice with DPBS. Adhesion was enabled with 180 μl overnight culture (8 × 10^8^–1.8 × 10^9^ cfu/ml) per well for 2 h at 37°C. The number of colony forming cells was determined by plating assay. Any non-adhered bacterial cells were removed from the wells by washing twice with DPBS and the wells were then dried for 20 min at 60°C. Dried and bound bacterial cells were stained with 100 μl crystal violet per well (Merck, Darmstadt, Germany) for 30 min at room temperature. Afterwards, the wells were washed with DPBS five times. The crystal violet was dissolved with 250-μl 70% ethanol per well with shaking (140 rpm) for 10 min at room temperature. The absorption of each well was measured using an Infinite m200 PRO plate reader (Tecan, Männedorf, Switzerland) with the following settings: 550 nm, 5 lightening, 2 x 2 in square. The experiment was performed on three different days with four technical replicates per day.

### Lysozyme- and hydrogen peroxide-resistance assay in terms of biofilm formation

Twenty microliters of bacterial culture (exponential phase; 4×10^8^–9×10^8^ cfu/ml) was added to 980 μl BHI medium in 24-well plates (culture plates, Greiner BioOne, Kremsmünster, Austria). The medium was either supplemented with 0, 10 or 20 mg/ml lysozyme from chicken egg white (Sigma, Steinheim, Germany) or with 0, 10 or 15 mM H_2_O_2_ (Roth, Karlsruhe, Germany). The inoculated medium was incubated at 37°C and 70 rpm [30]. Biofilm formation in the presence of H_2_O_2_ was only quantified after 5 h of incubation with crystal violet, whereas for the analysis of lysozyme treatment, bacteria were incubated for 5 h or 16 h. After incubation for 5 h, the supernatant with the non-adhered bacterial cells was removed and used for RNA extraction, see “cDNA synthesis from RNA of *S*. *gallolyticus* subsp. *gallolyticus*”. The wells were washed twice and adhered bacterial cells were either labelled for microscopic analysis or stained with crystal violet.

The number of viable bacteria was determined by degrading the biofilm with 1% saponin (Sigma, Steinheim, Germany) and performing a plating assay as described above. For microscopic analysis, bacterial DNA was labeled with 4′,6-diamidin-2-phenylindol (DAPI) for 30 min at room temperature, then wells were washed three times with DPBS and fixed with 4% formaldehyde for 30 min. Microscopy was performed with a Nikon Eclipse TE2000-S (Nikon instruments, Düsseldorf, Germany). Crystal violet staining was used for biofilm quantification as described above for collagen adhesion. The experiments were performed on four different days with four technical replicates per day.

### cDNA synthesis from RNA of *S*. *gallolyticus* subsp. *gallolyticus*

For transcriptome analysis of *S*. *gallolyticus* subsp. *gallolyticus* in response to lysozyme, 980 μl BHI medium with and without lysozyme (10 mg/ml) was inoculated with 20 μl bacterial culture in exponential phase. The cells were grown for 5 h in 24-well plates and RNA was extracted from planktonic cells.

Transcriptome analysis of collagen adhesion was performed as follows: bacterial cells from the exponential phase were cultivated in BHI in 12-well plates with or without immobilized collagen type I. After 2 h of incubation, planktonic cells (without collagen) were pelleted and cells with adhered collagen were washed once with DPBS. Microarray analysis was performed with three different biological replicates from three different days per condition. Real-time PCR was performed with samples from five different days with two technical replicates per day.

RNA was extracted with the peqGOLD Bacterial RNA Kit (VWR, Radnor, USA). Bacterial cells were suspended in TE buffer and lysis buffer T, transferred into Lysing Matrix B tubes (MP Biomedicals, Santa Ana, USA), and disrupted by using a Vortex-Genie 2 (Scientific Industries, New York, USA) for 3 min at full speed. Further RNA extraction was carried out following the manufacturer’s instructions. RNA was eluted with 30 μL RNase-free water and quantified using a NanoDrop 2000 (VWR, Radnor, USA). The RNA was used for microarray analysis and real-time PCR. For the latter, the synthesis of cDNA was carried out using the High-Capacity cDNA Reverse Transcription Kit (Thermo Scientific, Waltham, USA), following the manufacturer’s instructions. The cDNA was generated from 500 ng RNA by a one-step PCR. The cDNA was diluted in water at a ratio of 1:10 for real-time PCR.

### Gene expression analysis of *S*. *gallolyticus* subsp. *gallolyticus* using full-genome microarray

The microarrays had a customized design (MyArray; OakLabs GmbH, Hennigsdorf, Germany), which was generated out of four different *S*. *gallolyticus* subsp. *gallolyticus* genomes [31–35]. One array consists of 10,607 oligonucleotides targeting a total of 4,382 putative genes and non-annotated sequences. The cDNA and cRNA synthesis with Cy3 labelling and the microarray hybridization was carried out with the Quick Amp WT Labeling Kit, one-color (Agilent, Santa Clara, USA), following the manufacturer’s recommendations. The slides were washed and hybridization was stabilized with Stabilization and Drying solution (Agilent, Santa Clara, USA). After drying, the hybridized microarrays were scanned with a high-resolution Agilent microarray scanner G2565CA at a resolution of 5 μm and analyzed with the Feature extraction software (Agilent, Santa Clara, USA). Raw data were quantile-normalized and gene expression data were generated by the Direct Array software (OakLabs, Hennigsdorf, Germany). Statistical analysis was performed using Welch’s t-test. Thereby, all log_2_ values between -1 and 1 were not considered and only statistically significant values (*p*<0.05) are displayed. Raw data of the microarray results presented in this publication have been deposited in NCBI’s Gene Expression Omnibus and are accessible through GEO Series accession number GSE98955 (https://www.ncbi.nlm.nih.gov/geo/query/acc.cgi?acc=GSE98955; [36].

### Relative quantitative real-time PCR

Verification of *S*. *gallolyticus* subsp. *gallolyticus* microarray results were performed by real-time PCR on a LightCycler 480 II platform (Roche, Berlin, Germany). The reaction volume was 10 μl, containing 2.5 μL cDNA (dilution 1:10), 0.25 μL of each primer (20 μM; Table B in S1 File), 5.0 μL LightCycler 480 SYBR Green I Master-Kit (Roche, Berlin, Germany) and 2.0 μl water, and three replicates were run per sample. Denaturation of the reaction mix took place initially at 95°C (10 min) followed by 45 cycles consisting of denaturation for 10 s at 95°C, annealing at 65°C for 15 s and elongation at 72°C for 20 s. Additionally, a melting curve served as a control for PCR amplification. Relative gene expression was calculated by normalizing with reference genes by the efficiency-corrected ΔΔCt method [37]. To find the most stable reference genes, nine different possible reference genes were tested. geNorm determined the genes 16S rDNA and 23S rRNA (<1.5 C_t_ difference) as the ones with the most stable expression under both tested conditions (lysozyme/without lysozyme and collagen-adhered/planktonic). All oligonucleotides and their sequences are listed in Table B in S1 File.

### Statistics and *in silico* analysis

Experimental data were analyzed by Mann–Whitney *U* test using GraphPad Prism 6.0 (GraphPad Software, La Jolla, USA). *P* values less than 0.05 were considered statistically significant. Means with standard errors are displayed in the figures. DNA sequences were analyzed with clone manager (Scientific & Educational Software, Denver, USA) and PHAST [38], and protein function was determined with UniProt (EMBL-EBI, SIB and PIR, [39]).

## Results

### Lysozyme triggers biofilm formation of *S*. *gallolyticus* subsp. *gallolyticus*

**Fig 1:**
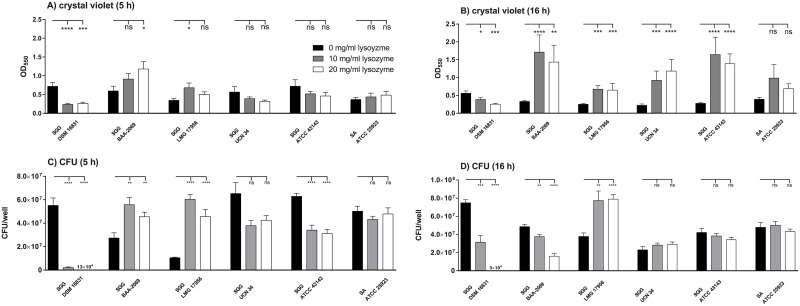
Biofilm formation after 5 and 16 h lysozyme treatment compared to the control. Biofilm formation on polystyrene was detected with crystal violet staining and absorption was determined photometrically (A+B) or by plaiting assay (C+D) after 5 and 16 h. The biofilm formation in BHI without lysozyme is compared to medium supplemented with lysozyme. Statistical significance between the different time points of a strain is marked with stars (Mann-Whitney *U* test, *: p < 0.05; **: p < 0.005; ****: p < 0.0001; n = 4). The standard deviation is marked with error bars. SGG = *Streptococcus gallolyticus* subsp. *gallolyticus*; SA = *Staphylococcus aureus*.

It has been shown that *S*. *gallolyticus* subsp. *gallolyticus* has a strain-dependent resistance to lysozyme [30], with strains building biofilms at the bottom of polystyrene wells when treated with lysozyme. Therefore, biofilm formation in the presence of lysozyme for five strains of *S*. *gallolyticus* subsp. *gallolyticus* and a strain of *S*. *aureus* serving as a control was quantified with crystal violet (Fig 1A+1B). It was revealed that biofilm formation of *S*. *gallolyticus* subsp. *gallolyticus* strain DSM 16831 decreased significantly with the addition of lysozyme after 5 and 16 h of incubation, independent of the used concentration. By contrast, the addition of 10 mg/ml lysozyme led to a significantly higher biofilm formation of *S*. *gallolyticus* subsp. *gallolyticus* strain LMG 17956, and addition of 20 mg/ml led to a significantly higher biofilm formation of *S*. *gallolyticus* subsp. *gallolyticus* strain BAA-2069 after 5 h of incubation. After 16 h, all strains of *S*. *gallolyticus* subsp. *gallolyticus*, except strain DSM 16831, showed higher biofilm formation. The control strain *S*. *aureus* ATCC 25923 showed no significant increase or decrease in biofilm formation at either time point. The same results were observed by microscopic analysis (Figure A in S1 File). Microscopic images revealed hardly any detectable bacterial cells of strain DSM 16831 after lysozyme treatment. The other strains aggregated and formed microcolonies as initial stages of biofilms. The determination of viable bacteria by plating assay revealed some different tendencies (Fig 1C+1D). After 5 h of incubation with lysozyme, less viable bacterial cells of strains DSM 16831 and ATCC 43143 were adhered to polystyrene compared to control, whereas more viable bacterial cells of strains BAA-2069 and LMG 17956 were found. After 16 h of incubation with lysozyme, the number of viable bacterial cells increased for strain LMG 17956 compared to control but the number of colony forming cells of strains BAA-2069 and DSM 16831 decreased.

It was shown that H_2_O_2_ can promote biofilm formation in other bacteria [3]. For *S*. *gallolyticus* subsp. *gallolyticus* it was shown that survival in H_2_O_2_-supplemented medium is strain dependent [30]. We performed a biofilm formation assay with H_2_O_2_ to analyze if this substance has the same effect as lysozyme on *S*. *gallolyticus* subsp. *gallolyticus* (Figure B in S1 File). The strains DSM 16831 and BAA-2069 formed a significantly lower amount of biofilms with 15 mM H_2_O_2_ compared to the control without H_2_O_2_. No significant increase or decrease in biofilm formation was observed for most strains, independent of the H_2_O_2_ concentration used. However, strains UCN 34 and ATCC 43143 tended to form more biofilm after 5 h in BHI containing 15 mM H_2_O_2_. Due to the observation that lysozyme has a considerably greater influence on biofilm formation compared to the treatment with H_2_O_2_, the transcriptome of *S*. *gallolyticus* subsp. *gallolyticus* was only analyzed in the presence of lysozyme.

### Transcriptome analysis of *S*. *gallolyticus* subsp. *gallolyticus* after lysozyme treatment

The transcriptomes of two strains were compared between planktonic bacterial cells grown in BHI and in BHI supplemented with 10 mg/ml lysozyme for 5 h. *S*. *gallolyticus* subsp. *gallolyticus* strain BAA-2069 showed a large increase in biofilm formation after lysozyme treatment. *S*. *gallolyticus* subsp. *gallolyticus* strain UCN 34 showed a high resistance against lysozyme [30]. Genes with different mRNA abundances under these conditions are listed in Table 1. The transcriptome analysis of strain BAA-2069 revealed 67 genes with increased expression in the presence of lysozyme, whereas no decreased gene expression was observed. By contrast, for strain UCN 34 the expression of 165 genes was increased in the presence of lysozyme and the expression of 30 genes was decreased. The largest group of affected genes were ribosomal genes; 23 ribosomal genes were differentially expressed in strain BAA-2069, and 32 ribosomal genes were differently expressed in strain UCN 34. Additionally, the expression of genes of protein-folding proteins and protein secretion (e.g., *prsA1* or *infA*), as well as genes of cell-wall synthesis (*dlt* operon) and immunity (*mccF*, *cinA*) were increased in both strains. Immunity proteins lead for example to resistance to microcins or increased DNA repair and thus reduce cell death [40,41]. A minimum of four fold-changes increase in gene expression was observed by 24% of the regulated genes in isolate BAA-2069. The genes *rpsR* encoding the 30S ribosomal protein S18 and *dltD* encoding the D-alanine extramembranal transfer protein were seven fold-changes higher expressed in the presence of lysozyme. Of the genes with increased expression in isolate UCN 34 in presence of lysozyme, 24% were also at least four times higher expressed. The gene of the polar amino acid transport system substrate-binding protein GALLO_0414 showed the highest increase in expression; it was 12.84-times higher in the presence of lysozyme compared to control. Additionally, the genes of the *dlt*-operon and of iron transporter binding proteins were 6.7–11.3 times higher expressed in strain UCN 34 in the presence of lysozyme treatment. The greatest decrease of gene expression was found for the Rrf2 family transcriptional regulators gene *GALLO_1840*, for which expression was 1.8-times reduced.

**Table 1:** Listed are genes which have different mRNA abundances in the *S*. *gallolyticus* subsp. *gallolyticus* strains BAA-2069 and UCN 34 in BHI supplemented with 10 mg/ml lysozyme compared to control (BHI without lysozyme) after 5 h of incubation.

Increased gene expression			BAA-2069			UCN 34		
function	gene	protein	log2 Ratio	p-value	fold-change	log2 Ratio	p-value	fold-change
antibiotic-resistance	*SGGBAA2069_c00120*	putative beta-lactamase	1.02	0.01	2.03	n. r.	-	n. r.
	*SGGBAA2069_c03220*	multiple antibiotic resistance protein marR	2.13	0.03	4.39	n. p.	-	n. p.
amino acid metabolism	*argG*	argininosuccinate synthase	n. r.	-	n. r.	1.59	0.02	3.01
	*asnA*	asparagine synthetase AsnA	1.06	0.01	2.09	n. r.	-	n. r.
	*citA*	citrate synthase	n. p.	-	n. p.	1.20	0.05	2.29
	*GALLO_0143*	acetyltransferase	n. r.	-	n. r.	1.09	0.02	2.13
	*GALLO_1269*	GNAT family acetyltransferase	n. p.	-	n. p.	1.34	0.01	2.52
	*GALLO_1848*	putative glutamine amidotransferase	n. p.	-	n. p.	2.28	0.01	4.87
	*gdh*	glutamate dehydrogenase	1.61	0.02	3.06	2.78	0.01	6.85
	*hipO1*	aminoacylase/N-acyl-L-amino acid amidohydrolase/hippurate hydrolase	n. r.	-	n. r.	1.57	0.00	2.98
	*nifS*	cysteine desulfurase/ aminotransferase	1.28	0.01	2.42	1.35	0.02	2.55
	*panE*	2-dehydropantoate 2-reductase	n. r.	-	n. r.	1.46	0.04	2.76
	*SGGBAA2069_c18080*	putative glutamine amidotransferase	1.99	0.01	3.97	n. r.	-	n. r.
	*sufS*	cysteine desulfurase / selenocysteine lyase	n. r.	-	n. r.	1.17	0.00	2.24
carbohydrate metabolism	*gpmA*	phosphoglyceromutase	n. r.	-	n. r.	1.33	0.01	2.51
	*icd*	isocitrate dehydrogenase	n. r.	-	n. r.	1.07	0.02	2.10
	*pfkA*	6-phosphofructokinase	n. r.	-	n. r.	1.48	0.03	2.78
	*pgi*	glucose-6-phosphate isomerase	n. r.	-	n. r.	1.01	0.02	2.01
	*pyk*	pyruvate kinase	n. r.	-	n. r.	1.14	0.04	2.21
DNA-binding/ repair	*GALLO_0671*	phosphoglycolate phosphatase	n. r.	-	n. r.	1.33	0.00	2.52
	*GALLO_0742*	DHH family phosphatase protein	n. r.	-	n. r.	1.27	0.01	2.41
	*ogt*	6-O-methylguanine DNA methyltransferase	1.02	0.04	2.02	1.70	0.01	3.26
	*parC*	topoisomerase IV subunit A	n. r.	-	n. r.	1.18	0.04	2.27
	*pelL1*	pectate lyase L	2.04	0.03	4.10	1.29	0.04	2.45
	*polC*	DNA polymerase III	n. r.	-	n. r.	1.22	0.04	2.33
	*recO*	DNA repair protein recO	1.10	0.02	2.15	n. r.	-	n. r.
	*rggA*	putative transcriptional activator Rgg/GadR/MutR	n. p.	-	n. p.	1.54	0.03	2.91
	*ssbA*	single-stranded DNA-binding protein	n. p.	-	n. p.	2.52	0.02	5.74
	*ssbB*	single-strand DNA-binding protein	2.36	0.02	5.12	n. r.	-	n. r.
	*topA*	DNA topoisomerase I	n. r.	-	n. r.	1.86	0.04	3.64
fatty acid metabolism	*accA*	acetyl-CoA carboxylase subunit alpha	n. r.	-	n. r.	1.74	0.00	3.34
	*accB*	acetyl-CoA carboxylase biotin carboxyl carrier protein subunit	n. r.	-	n. r.	2.25	0.01	4.76
	*accC*	acetyl-CoA carboxylase biotin carboxylase subunit	n. r.	-	n. r.	1.74	0.02	3.35
	*accD*	acetyl-CoA carboxylase subunit beta	n. r.	-	n. r.	1.53	0.04	2.89
	*fabD*	malonyl CoA-acyl carrier protein transacylase	n. r.	-	n. r.	2.12	0.02	4.36
	*fabF*	3-oxoacyl-(acyl carrier protein) synthase II	n. r.	-	n. r.	1.98	0.00	3.94
	*fabG*	3-ketoacyl-ACP reductase	n. r.	-	n. r.	2.31	0.01	4.95
	*fabH*	3-oxoacyl-ACP synthase	1.75	0.02	3.36	2.27	0.00	4.82
	*fabK*	enoyl-(acyl-carrier-protein) reductase II	1.45	0.05	2.74	2.27	0.00	4.81
	*fabZ*	(3R)-hydroxymyristoyl-ACP dehydratase	n. r.	-	n. r.	1.95	0.00	3.87
	*GALLO_0333*	enoyl-CoA hydratase	n.r	-	n. r.	2.46	0.01	5.50
	*GALLO_0975*	glycerol-3-phosphate acyltransferase PlsY	n. p.	-	n. p.	1.06	0.02	2.08
	*phaB*	enoyl-CoA hydratase	2.28	0.03	4.85	2.17	0.00	4.50
GMP biosynthesis	*guaB*	inosine 5’-monophosphate dehydrogenase	n. r.	-	n. r.	1.42	0.02	2.67
hydrogen peroxid-reduction	*ahpC*	alkyl hydroperoxide reductase	1.17	0.02	2.25	1.90	0.01	3.74
	*ahpF*	alkyl hydroperoxide reductase subunit F	1.07	0.04	2.10	1.89	0.04	3.70
	*naoX*	NADH oxidase	1.05	0.03	2.07	n. p.	-	n. p.
immunity	*mccF*	microcin immunity protein MccF	1.67	0.03	3.18	2.22	0.00	4.65
	*cinA*	competence damage-inducible protein A	1.34	0.05	2.53	2.00	0.02	4.00
metabolism	*phnA*	phosphonoacetate hydrolase	n. r.	-	n. r.	1.38	0.05	2.60
metal binding	*GALLO_0832*	metal dependent phosphohydrolase	n. p.	-	n. p.	1.59	0.02	3.00
nucleotide biosthesis	*prs*	ribose-phosphate pyrophosphokinase	n. r.	-	n. r.	1.19	0.02	2.28
	*add*	adenosine deaminase	n. r.	-	n. r.	1.08	0.04	2.12
oxireductase	*gapN*	NADP-dependent glyceraldehyde-3-phosphate dehydrogenase	n. r.	-	n. r.	1.91	0.02	3.76
phage protein	*int5*	site-specific recombinase, phage integrase family	n. r.	-	n. r.	1.10	0.03	2.15
porphyrin synthesis	*GALLO_1275*	uroporphyrinogen decarboxylase	n. p.	-	n. p.	1.97	0.01	3.92
protease	*clpP*	ATP-dependent Clp protease proteolytic subunit	n. r.	-	n. r.	1.08	0.03	2.11
	*GALLO_0849*	Zn-dependent protease	n. p.	-	n. p.	2.02	0.05	4.05
	*GALLO_2250*	insulinase, M16 family peptidase	n. r.	-	n. r.	1.02	0.04	2.02
	*pepB*	oligoendopeptidase F	n. r.	-	n. r.	1.31	0.03	2.48
	*pepO*	putative endopeptidase	n. r.	-	n. r.	1.29	0.02	2.44
protein secretion/synthesis	*alaS*	alanyl-tRNA ligase	n. r.	-	n. r.	1.29	0.03	2.45
	*asnA*	asparagine synthetase AsnA	n. r.	-	n. r.	1.70	0.03	3.26
	*cysS*	cysteinyl-tRNA synthetase	n. r.	-	n. r.	1.49	0.01	2.80
	*GALLO_1298*	queuosine biosynthesis protein	n. r.	-	n. r.	2.21	0.05	4.63
	*GALLO_1812*	RNA methyltransferase	n. p.	-	n. p.	1.86	0.00	3.64
	*gatA*	aspartyl/glutamyl-tRNA amidotransferase subunit A	n. r.	-	n. r.	1.31	0.01	2.47
	*ileS*	isoleucyl-tRNA ligase	n. r.	-	n. r.	1.24	0.02	2.36
	*infA*	translation initiation factor IF-1	2.00	0.03	4.01	1.69	0.00	3.23
	*leuS*	leucyl-tRNA synthetase	n. r.	-	n. r.	1.07	0.02	2.09
	*prsA1*	foldase protein PrsA	1.84	0.01	3.58	2.12	0.04	4.35
	*thrS*	threonyl-tRNA ligase	n. r.	-	n. r.	1.37	0.00	2.59
	*tig*	trigger factor	1.11	0.01	2.16	n. r.	-	n. r.
	*trmF*	tRNA uridine 5-carboxymethylaminomethyl modification enzyme	n. r.	-	n. r.	1.12	0.02	2.18
	*mnmA*	tRNA-specific 2-thiouridylase MnmA	1.21	0.01	2.31	1.54	0.02	2.91
	*tyrS*	tyrosyl-tRNA synthetase	n. r.	-	n. r.	1.26	0.03	2.40
	*yqaB*	acetyltransferase	n. r.	-	n. r.	1.27	0.02	2.41
proton transport	*atpB*	F0F1 ATP synthase subunit A	1.40	0.03	2.64	n. r.	-	n. r.
	*atpF*	F0F1 ATP synthase subunit B	1.47	0.01	2.76	n. r.	-	n. r.
redox metabolism	*gor*	glutathione reductase	n. r.	-	n. r.	1.57	0.02	2.97
arsenate resistance	*GALLO_1741*	arsenate reductase family protein	n. r.	-	n. r.	1.14	0.01	2.21
ribosome	*prfC*	peptide chain release factor 3	1.32	0.01	2.49	1.48	0.02	2.78
	*rplA*	50S ribosomal protein L1	1.89	0.02	3.70	2.37	0.02	5.18
	*rplC*	50S ribosomal protein L3	1.88	0.01	3.68	2.13	0.04	4.38
	*rplD*	50S ribosomal protein L4	1.47	0.03	2.78	1.97	0.01	3.91
	*rplE*	50S ribosomal protein L5	1.05	0.04	2.07	n. r.	-	n. r.
	*rplF*	50S ribosomal protein L6	1.03	0.04	2.04	1.36	0.03	2.56
	*rplJ*	50S ribosomal protein L10	1.81	0.03	3.51	2.39	0.00	5.24
	*rplK*	50S ribosomal protein L11	2.35	0.01	5.10	2.62	0.05	6.15
	*rplL*	50S ribosomal protein L7/L12	1.65	0.01	3.14	n. r.	-	n. r.
	*rplM*	50S ribosomal protein L13	n. r.	-	n. r.	1.39	0.03	2.62
	*rplN*	50S ribosomal protein L14	1.12	0.03	2.17	n. r.	-	n. r.
	*rplO*	50S ribosomal protein L15	1.53	0.03	2.89	1.63	0.04	3.09
	*rplP*	50S ribosomal protein L16	1.22	0.05	2.33	1.59	0.04	3.02
	*rplQ*	50S ribosomal protein L17	n. r.	-	n. r.	1.48	0.02	2.80
	*rplU*	50S ribosomal protein L21	1.28	0.02	2.42	1.75	0.00	3.36
	*rplV*	50S ribosomal protein L22	1.48	0.03	2.78	1.53	0.01	2.88
	*rplW*	50S ribosomal protein L23	1.46	0.02	2.75	1.69	0.00	3.22
	*rplX*	50S ribosomal protein L24	n. r.	-	n. r.	1.48	0.01	2.79
	*rpmC*	50S ribosomal protein L29	1.34	0.04	2.53	1.43	0.00	2.69
	*rpmD*	50S ribosomal protein L30	1.51	0.01	2.84	n. r.	-	n. r.
	*rpmJ*	50S ribosomal protein L36	n. r.	-	n. r.	1.52	0.02	2.88
	*rpmQ*	50S ribosomal protein L30	n. r.	-	n. r.	1.28	0.03	2.43
	*rpsB*	30S ribosomal protein S2	1.61	0.04	3.06	2.30	0.00	4.92
	*rpsC*	30S ribosomal protein S3	n. r.	-	n. r.	1.58	0.03	3.00
	*rpsD*	30S ribosomal protein S4	n. r.	-	n. r.	1.26	0.02	2.39
	*rpsE*	30S ribosomal protein S5	1.19	0.04	2.28	1.43	0.02	2.69
	*rpsF*	30S ribosomal protein S6	1.97	0.01	3.93	2.39	0.01	5.26
	*rpsG*	30S ribosomal protein S7	1.23	0.01	2.35	1.26	0.00	2.40
	*rpsH*	30S ribosomal protein S8	n. r.	-	n. r.	1.12	0.00	2.18
	*rpsI*	30S ribosomal protein S9	n. r.	-	n. r.	1.17	0.02	2.25
	*rpsJ*	30S ribosomal protein S10	1.95	0.03	3.86	2.00	0.04	4.01
	*rpsK*	30S ribosomal protein S11	n. r.	-	n. r.	1.51	0.02	2.85
	*rpsM*	30S ribosomal protein S13	n. r.	-	n. r.	1.78	0.05	3.45
	*rpsQ*	30S ribosomal protein S17	1.13	0.04	2.19	1.54	0.00	2.91
	*rpsR*	30S ribosomal protein S18	2.79	0.01	6.93	n. r.	-	n. r.
	*rpsS*	30S ribosomal protein S19	n. r.	-	n. r.	1.72	0.02	3.28
RNA biogenesis	*cysS*	cysteinyl-tRNA synthetase	1.11	0.01	2.16	n. r.	-	n. r.
	*SGGBAA2069_c02420*	hypothetical protein/putative ribonuclease III family protein	1.16	0.01	2.23	n. r.	-	n. r.
	*tilS*	tRNA(Ile)-lysidine synthetase	1.48	0.04	2.79	n. r.	-	n. r.
nitrogen balance	*gnlB*	nitrogen regulatory protein PII	1.85	0.01	3.62	n. p.	-	n. p.
	*GALLO_1945*	nitroreductase	n. p.	-	n. p.	1.50	0.01	2.83
	*yqaB*	acetyltransferase	n. r.	-	n. r.	1.47	0.01	2.77
transcription	*GALLO_0107*	3-demethylubiquinone-9 3-methyltransferase	n. r.	-	n. r.	1.64	0.02	3.11
	*rpoA*	DNA-directed RNA polymerase subunit alpha	n. r.	-	n. r.	1.78	0.04	3.44
	*tsf*	elongation factor Ts	n. r.	-	n. r.	1.74	0.04	3.34
thiamine biosynthesis	*pdxK*	phosphomethylpyrimidine kinase	n. r.	-	n. r.	1.41	0.01	2.66
transporter	*atmB*	ABC transporter ATP-binding protein	1.66	0.01	3.17	2.08	0.03	4.24
	*atpA*	F-type H+-transporting ATPase subunit alpha	n. r.	-	n. r.	1.52	0.04	2.88
	*atpB*	F0F1 ATP synthase subunit A	1.40	0.03	2.64	2.40	0.02	5.26
	*atpE*	F0F1 ATP synthase subunit C	n. r.	-	n. r.	1.80	0.01	3.49
	*atpF*	F0F1 ATP synthase subunit B	1.47	0.01	2.76	2.27	0.01	4.82
	*atpH*	F0F1 ATP synthase subunit delta	n. r.	-	n. r.	2.10	0.00	4.27
	*cysA*	sulfate/thiosulfate import ATP-binding protein cysA	n. r.	-	n. r.	1.06	0.05	2.09
	*fhuC*	iron complex transport system ATP-binding protein	n. r.	-	n. r.	2.99	0.01	7.92
	*fhuD*	iron (Fe+3) ABC transporter binding protein	n. r.	-	n. r.	2.93	0.02	7.63
	*GALLO_0402*	ABC transporter ATP-binding protein	n. r.	-	n. r.	1.42	0.03	2.68
	*GALLO_0414*	polar amino acid transport system substrate-binding protein	n. p.	-	n. p.	3.68	0.01	12.84
	*GALLO_0415*	amino acid ABC transporter membrane protein	n. p.	-	n. p.	2.07	0.00	4.19
	*GALLO_0902*	N-acetyltransferase GCN5	n. r.	-	n. r.	2.05	0.01	4.14
	*GALLO_1167*	cobalt/nickel transport system ATP-binding protein	n. r.	-	n. r.	1.25	0.04	2.38
	*GALLO_1168*	cobalt/nickel transport system permease protein	n. p.	-	n. p.	1.23	0.02	2.34
	*GALLO_1269*	N-acetyltransferase GCN5	n. p.	-	n. p.	1.23	0.00	2.35
	*GALLO_1301*	ABC transporter ATP-binding protein	n. p.	-	n. p.	1.48	0.03	2.79
	*GALLO_1745*	GNAT family acetyltransferase	n. p.	-	n. p.	1.09	0.02	2.12
	*GALLO_1845*	polar amino acid transport system substrate-binding protein	n. p.	-	n. p.	2.83	0.04	7.12
	*GALLO_1847*	amino acid ABC transporter substrate-binding protein	n. p.	-	n. p.	1.51	0.00	2.84
	*proB*	ABC transporter permease	n. r.	-	n. r.	1.48	0.01	2.79
	*ptsB*	phosphate import ATP-binding protein pstB	n. p.	-	n. p.	1.12	0.02	2.17
	*sufD*	iron-sulfur ABC transporter	n. r.	-	n. r.	1.40	0.03	2.65
	*yjgC*	amino acid ABC transporter substrate binding protein	n. r.	-	n. r.	1.39	0.00	2.62
	*ytmK*	amino acid ABC transporter permease	1.00	0.01	2.01	1.66	0.04	3.16
	*rpsJ*	polar amino acid transport system substrate-binding protein	1.94	0.05	3.83	n. r.	-	n. r.
	*SGGBAA2069_c04070*	polar amino acid transport system substrate-binding protein	2.64	0.00	6.22	n. p.	-	n. p.
	*SGGBAA2069_c04080*	amino acid ABC transporter membrane protein	1.58	0.01	2.99	n. p.	-	n. p.
	*SGGBAA2069_c04090*	polar amino acid transport system ATP-binding protein	1.52	0.04	2.86	n. p.	-	n. p.
	*SGGBAA2069_c18050*	polar amino acid transport system substrate-binding protein	2.19	0.00	4.57	n. r.	-	n. r.
unknown function	*GALLO_0309*	hypothetical protein	n. r.	-	n. r.	1.18	0.01	2.26
	*GALLO_0353*	membrane protein	n. r.	-	n. r.	2.77	0.02	6.84
	*GALLO_0481*	hypothetical protein	n. p.	-	n. p.	1.01	0.05	2.02
	*GALLO_0527*	hypothetical protein	n. p.	-	n. p.	1.20	0.01	2.30
	*GALLO_0624*	hypothetical protein	n. p.	-	n. p.	2.70	0.02	6.48
	*GALLO_0742*	phosphoesterase	n. r.	-	n. r.	1.17	0.01	2.25
	*GALLO_0855*	hypothetical protein	n. r.	-	n. r.	1.14	0.03	2.20
	*GALLO_0975*	hypothetical protein	n. p.	-	n. p.	1.25	0.04	2.37
	*GALLO_1073*	hypothetical protein	n. p.	-	n. p.	1.16	0.01	2.23
	*GALLO_1171*	ATP-binding protein	n. r.	-	n. r.	1.59	0.01	3.00
	*GALLO_1176*	hypothetical protein	n. r.	-	n. r.	1.02	0.03	2.03
	*GALLO_1275*	hypothetical protein	n. p.	-	n. p.	1.84	0.02	3.59
	*GALLO_1342*	hypothetical protein	n. p.	-	n. p.	1.63	0.00	3.10
	*GALLO_1559*	membrane protein	n. r.	-	n. r.	1.26	0.03	2.40
	*GALLO_1637*	hypothetical protein	n. r.	-	n. r.	1.67	0.01	3.17
	*GALLO_1877*	aminotransferase AlaT	n. p.	-	n. p.	1.03	0.05	2.04
	*GALLO_2085*	hypothetical protein	n. r.	-	n. r.	2.38	0.02	5.19
	*GALLO_2086*	hypothetical protein	n. r.	-	n. r.	1.31	0.03	2.48
	*SGGBAA2069_c02390/GALLO_0224*	hypothetical protein	1.24	0.04	2.36	1.42	0.04	2.67
	*SGGBAA2069_c06260*	hypothetical protein	1.80	0.01	3.49	n. r.	-	n. r.
	*SGGBAA2069_c07890*	hypothetical protein	1.99	0.01	3.96	n. r.	-	n. r.
	*SGGBAA2069_c12660*	hypothetical protein	1.71	0.02	3.28	n. p.	-	n. p.
	*SGGBAA2069_c13310*	hypothetical protein	1.36	0.04	2.57	n. p.	-	n. p.
cell wall/cell division	*dltA*	D-alanine—poly(phosphoribitol) ligase subunit 1	1.18	0.02	2.27	n. r.	-	n. r.
	*dltB*	D-alanine transfer protein DltB	2.16	0.01	4.46	3.25	0.02	9.48
	*dltC*	D-alanine—poly(phosphoribitol) ligase subunit 2	1.98	0.02	3.94	3.16	0.04	8.93
	*dltD*	D-alanine extramembranal transfer protein	2.77	0.01	6.83	3.51	0.02	11.36
	*ftsH*	cell division protein FtsH	1.14	0.05	2.20	1.59	0.01	3.01
	*lss*	N-acetylmuramidase/lysin	n. r.	-	n. r.	2.00	0.03	4.01
	*murB*	UDP-N-acetylenolpyruvoylglucosamine reductase	n. r.	-	n. r.	1.31	0.01	2.47
	*rmlA*	glucose-1-phosphate thymidylyltransferase	n. r.	-	n. r.	1.18	0.05	2.27
	*rmlC*	dTDP-4-dehydrorhamnose 3,5-epimerase	n. r.	-	n. r.	1.22	0.03	2.33
Decreased gene expression			BAA-2069			UCN 34		
Function	Gene	protein	log2 Ratio	p-value	fold-change	log2 Ratio	p-value	fold-change
acid tolerance	*satD*	putative secretion and acid tolerance protein SatD	n. r.	-	n. r.	-1.08	0.02	0.47
antibiotic resistance	*norN*	Multidrug resistance protein mdtK	n. r.	-	n. r.	-1.03	0.05	0.49
	*GALLO_0083*	penicillin binding protein 1B	n. r.	-	n. r.	-1.05	0.02	0.48
carbohydrate metabolism	*bglA*	beta-glucosidase	n. r.	-	n. r.	-1.44	0.03	0.37
competence	*cglA*	putative competence protein	n. r.	-	n. r.	-1.39	0.04	0.38
	*comEA*	exogenous DNA-binding protein	n. r.	-	n. r.	-1.22	0.01	0.43
	*GALLO_0088*	putative competence protein, ABC transporter	n. r.	-	n. r.	-1.02	0.02	0.49
DNA binding	*GALLO_1840*	Rrf2 family transcriptional regulators	n. p.	-	n. p.	-1.76	0.00	0.30
	*GALLO_0923*	LysR family transcriptional regulator	n. p.	-	n. p.	-1.45	0.04	0.37
	*rggB*	transcriptional regulator	n. p.	-	n. p.	-1.43	0.03	0.37
DNA repair	*GALLO_1079*	AraC family transcriptional regulator	n. r.	-	n. r.	-1.56	0.03	0.34
Hydrogen peroxide resistance	*dpr*	peroxide resistance protein Dpr	n. r.	-	n. r.	-1.07	0.04	0.48
protease	*GALLO_1986*	putative O-sialoglycoprotein endopeptidase	n. p.	-	n. p.	-1.00	0.04	0.50
protein synthesis	*miaA*	tRNA delta(2)-isopentenylpyrophosphate transferase	n. r.	-	n. r.	-1.32	0.02	0.40
	*GALLO_0368*	ribosome maturation protein RimP	n. r.	-	n. r.	-1.38	0.04	0.38
ribosome	*thiE*	thiamine-phosphate pyrophosphorylase	n. r.	-	n. r.	-1.09	0.05	0.47
thiamine biosynthesis	*frwB*	PTS system fructose-specific transporter subunit IIB	n. r.	-	n. r.	-1.52	0.05	0.35
transporter	*GALLO_1155*	zinc transporter, ZIP family	n. p.	-	n. p.	-1.01	0.02	0.49
	*GALLO_2102*	CHY zinc finger family protein	n. r.	-	n. r.	-1.46	0.03	0.36
unknown function	*GALLO_1644*	hypothetical protein	n. r.	-	n. r.	-1.37	0.03	0.39
	*GALLO_2018*	cell wall associated protein (LPXTG motive)	n. r.	-	n. r.	-1.25	0.05	0.42
	*GALLO_1799*	hypothetical protein	n. r.	-	n. r.	-1.22	0.04	0.43
	*GALLO_0091*	hypothetical protein	n. r.	-	n. r.	-1.22	0.04	0.43
	*GALLO_0141*	hypothetical protein	n. r.	-	n. r.	-1.20	0.03	0.44
	*GALLO_0915*	hypothetical protein	n. p.	-	n. p.	-1.13	0.03	0.46
	*GALLO_1780*	hypothetical protein	n. p.	-	n. p.	-1.12	0.01	0.46
	*GALLO_0718*	hypothetical protein	n. p.	-	n. p.	-1.07	0.02	0.48
	*GALLO_1493*	hypothetical protein	n. r.	-	n. r.	-1.06	0.04	0.48
	*GALLO_1207*	short chain dehydrogenase	n. r.	-	n. r.	-1.04	0.02	0.49
	*GALLO_1447*	hypothetical protein	n. r.	-	n. r.	-1.02	0.04	0.49

n = 3; n. r. = no differences in mRNA abundances; n. p. = gene not present in respective strain; - = not relevant

**Fig 2:**
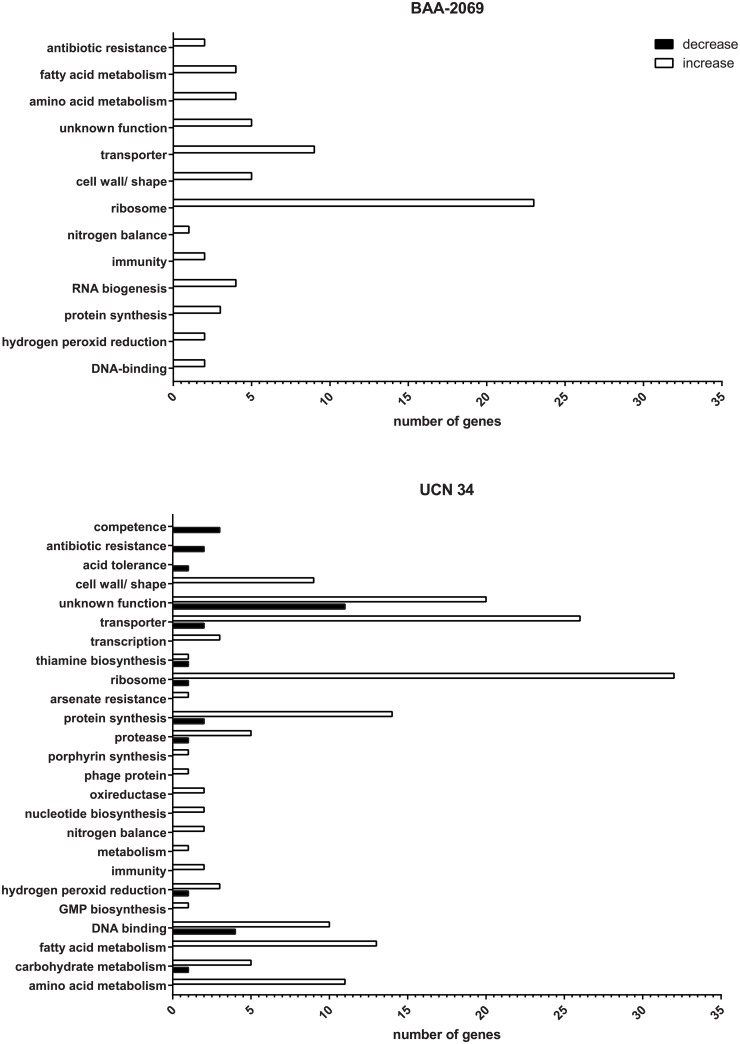
The number of regulated genes determined by microarray analysis. The number of genes which were regulated in the *S*. *gallolyticus* subsp. *gallolyticus* strains BAA-2069 and UCN 34 after lysozyme treatment for 5 h (black: decrease, white: increased) are displayed on the x-axis. Genes were sorted into functional categories.

Although the expression of ribosomal genes was strongly increased through lysozyme treatment, the abundances of ribosomal RNA did not differ between control and lysozyme-treated bacteria. Therefore, it was possible to use 16S and 23S rRNA genes as references for relative quantitative real-time PCR. Additionally, genes involved in different metabolic pathways, such as amino acid, carbohydrate and fatty acid metabolism, had increased expression compared to controls. Lysozyme treatment also triggers higher expression of genes which are involved in peroxide metabolism (Fig 2). Genes of competence systems, acid and antibiotic tolerance, and a few genes of metabolism were downregulated due to lysozyme treatment in strain UCN 34.

### Verification of microarray results for lysozyme treatment by relative quantitative real-time PCR

**Fig 3:**
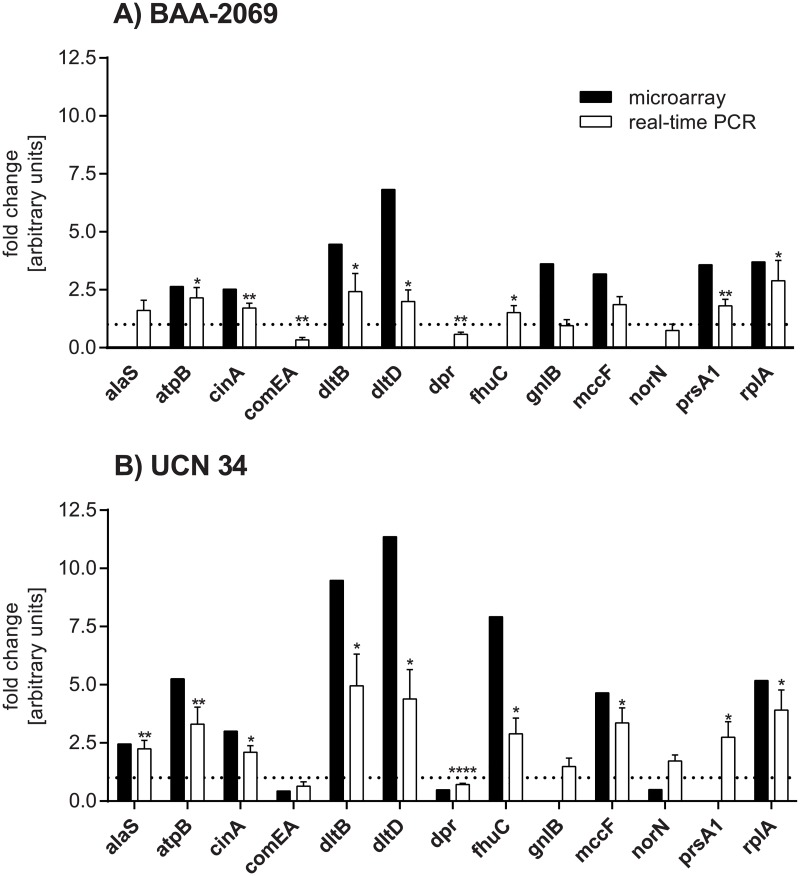
Verification of gene expression changes determined by microarray analysis of lysozyme-treated bacterial cells with relative quantitative real-time PCR. The fold change of the regulation of distinct genes (x-axis) identified by microarray analysis (black) and real-time PCR (white) is displayed for BAA-2069 (A) and UCN 34 (B). The dotted line represents the relative mRNA level in the control which is set by one. Statistical significance between the control mRNA abundances (set as one; dotted line) and the mRNA abundances of lysozyme treated cells are marked with stars (Mann-Whitney *U* test, *: p < 0.05; **: p < 0.005; ****: p < 0.0001; n = 8). n.d. = not detected.

Relative quantitative real-time PCR was used to verify the microarray results, analyzing genes of interest such as virulence- and immunity-associated genes (Fig 3). The real-time PCR analysis resulted in lower changes in gene expression than the microarray analysis. Nevertheless, changes in gene expression found by microarray analysis could be confirmed by real-time PCR. Exceptions are the decrease of *comEA* expression which could not be verified in strain UCN 34 by real-time PCR, but a decrease in gene expression was shown in strain BAA-2069. Additionally, the decrease of *norN* expression could not be verified for strain UCN 34 by real-time PCR.

### Adhesion of *S*. *gallolyticus* subsp. *gallolyticus* to collagen type I and IV

**Fig 4:**
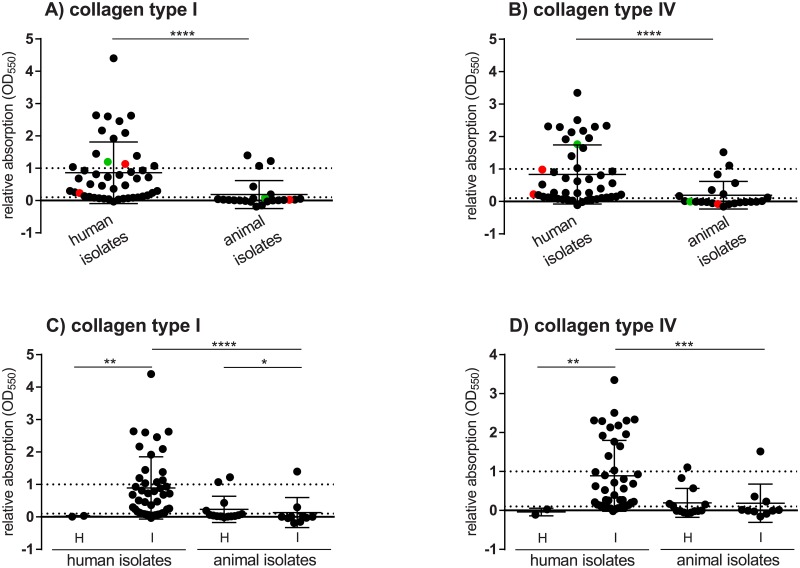
Adhesion of *S*. *gallolyticus* subsp. *gallolyticus* to collagen type I and IV. Adhesion to collagen type I (A+C) and IV (B+D) was detected with crystal violet and absorption was determined photometrically after 2 h. Compared are isolates which originated from humans and animals. Additionally, strains which are associated with infections and which were isolated from healthy probands or animals have been compared (C+D). The strains DSM 16831, BAA-2069, LMG 17956, UCN 34 and ATCC43143, which were used in the lysozyme biofilm formation test, are highlighted in red (BAA-2069, LMG 17956 and ATCC 43143) and green (UCN 34 and DSM 16831). Strains are divided into high adhesion ability (> 1), medium adhesion ability (0.1–1) and low or no adhesion ability (< 0.1). Mean with standard error is shown. Statistical significance between the groups of *S*. *gallolyticus* subsp. *gallolyticus* isolates are marked with stars (Mann-Whitney *U* test, *: p < 0.05; **: p < 0.005; ***: p < 0.0005; ****: p < 0.0001; n = 3 per isolate). H = isolates from healthy probands/animals; I = infection-associated isolates.

Collagen-dependent adhesion and biofilm formation are relevant virulence mechanisms of bacteria for establishing IE. Therefore, we analyzed strain-dependent adhesion to collagen type I and collagen type IV compared to BSA. The adhesion to BSA served as negative control and was subtracted from the adhesion to collagen type I or IV of each isolate, respectively. The adhesion ability of human isolates was compared to that of isolates from animals (Fig 4). Strains could be classified into three different categories: low adhesion (<0.1), medium adhesion (0.1–1) and high adhesion (>1). Strains of *S*. *gallolyticus* subsp. *gallolyticus* isolated from human patients show significantly higher adhesion to both tested types of collagen than strains isolated from animals (Fig 4A+4B), regardless of whether the strains are associated with infections or isolated from healthy organisms (Fig 4C+4D). In Figure C in S1 File, the adhesion ability of each of the 69 human and animal isolates is shown. Additionally, isolates from other origins and three other bacterial species–*S*. *aureus*, *Escherichia coli* and *Lactococcus lactis*–were tested. The adhesion capability of the five strains represented by highlighted red and green dots in Fig 4, whose biofilm formation in the presence of lysozyme was analyzed, are subsequently described. When comparing the five strains, strain UCN 34 had the highest ability to adhere to collagen type IV and type I. Strains DSM 16831 and LMG 17956 could only adhere to collagen type I; no adhesion was observed for collagen type IV (<0). The isolate ATCC 43143 adhered slightly less to collagen than strain UCN 34, and strain BAA-2069 showed only medium adhesion ability.

The influence of lysozyme treatment on adhesion to collagen type I (Figure D in S1 File) was also analyzed. It was observed that lysozyme led to a significantly higher adhesion of strain BAA-2069 to BSA and collagen type I after incubation for 5 h. The adhesion ability of the other *S*. *gallolyticus* subsp. *gallolyticus* strains tested and *S*. *aureus* strain ATCC 25923 were not influenced by lysozyme treatment after 5 h.

### Transcriptome analysis of adhered *S*. *gallolyticus* subsp. *gallolyticus* to collagen type I

Transcriptome analysis was performed to determine differences in mRNA abundances between planktonic and collagen-type-I-adhered bacterial cells, both in BHI medium. Thereby, two strains were analyzed because they showed noticeably different collagen adhesion abilities. Strain UCN 34 adhered strongly to collagen types I and IV, whereas strain DSM 16831 adhered only marginally to collagen type I (Fig 4, green dots). Transcriptome analysis revealed that both strains showed divergent gene expression profiles by collagen adhesion (Table 2). Genes of two regions showed an increase in expression in the genome of *S*. *gallolyticus* subsp. *gallolyticus* strain DSM 16831. One region consists of 15 phage-associated genes and the other of 25 genes which belong to an integrative and conjugative element (ICE). *In silico* analysis with PHAST revealed that the phage genes belong to a 49.8-kb complete bacteriophage region which has high similarity with the *Streptococcus* phage P9 (NC_009819) [38]. In *S*. *gallolyticus* subsp. *gallolyticus* strain UCN 34, the expression of 27 genes was upregulated after incubation for 2 h with collagen type I, whereas the expression of 31 genes was downregulated. Genes of transporters showed mostly an increase in gene expression, while the expression of genes which belong to metabolism pathways of carbohydrates and lipids were decreased.

**Table 2:** Listed are genes with different mRNA in the *S*. *gallolyticus* subsp. *gallolyticus* strains DSM 16831 or UCN 34 in BHI adhered to collagen compared to control (planktonic bacterial cells in BHI without collagen).

DSM 16831: Increased gene expression					
function	gene	protein	log2 Ratio	p-value	fold-change
Phage proteins	*BTR42_02550*	phage head protein	1.10	0.02	2.15
	*BTR42_02555*	phage protein	1.43	0.02	2.69
	*BTR42_02560*	phage protein	1.36	0.02	2.58
	*BTR42_02565*	phage protein	1.03	0.02	2.05
	*BTR42_02570*	phage protein	1.26	0.01	2.40
	*BTR42_02575*	phage protein	1.12	0.05	2.17
	*BTR42_02580*	phage protein	1.02	0.03	2.03
	*BTR42_02585*	phage protein	1.11	0.02	2.16
	*BTR42_02595*	phage protein	1.28	0.02	2.43
	*BTR42_02600*	phage protein	1.16	0.04	2.23
	*BTR42_02605*	phage protein	1.08	0.01	2.12
	*BTR42_02610*	phage protein	1.14	0.03	2.20
	*BTR42_02615*	phage protein	1.28	0.03	2.42
	*BTR42_02620*	hypothetical protein	1.38	0.03	2.61
	*BTR42_02630*	hypothetical protein	1.23	0.02	2.34
transposon	*BTR42_07290*	relaxase	1.11	0.01	2.16
	*BTR42_07295*	mobilisation protein	1.35	0.00	2.55
	*BTR42_07305*	hypothetical protein	1.10	0.00	2.14
	*BTR42_07320*	putative transcriptional regulator	1.42	0.03	2.68
	*BTR42_07335*	conjugative transposon protein	1.71	0.02	3.27
	*BTR42_07340*	hypothetical protein	1.35	0.04	2.55
	*BTR42_07355*	hypothetical protein	1.67	0.03	3.19
	*BTR42_07360*	hypothetical protein	1.85	0.01	3.59
	*BTR42_07365*	hypothetical protein	1.29	0.02	2.45
	*BTR42_07370*	hypothetical protein	1.47	0.03	2.77
	*BTR42_07375*	hypothetical protein	1.61	0.03	3.05
	*BTR42_07380*	hypothetical protein	1.61	0.01	3.05
	*BTR42_07390*	hypothetical protein	1.38	0.02	2.60
	*BTR42_07400*	hypothetical protein	1.30	0.03	2.47
	*BTR42_07410*	hypothetical protein	1.25	0.01	2.38
	*BTR42_07435*	extracellular protein	1.53	0.04	2.89
	*BTR42_07440*	hypothetical protein	1.93	0.03	3.81
	*BTR42_07445*	hypothetical protein	1.45	0.03	2.73
	*BTR42_07450*	hypothetical protein	1.33	0.03	2.52
	*BTR42_07455*	hypothetical protein	1.05	0.04	2.08
	*BTR42_07460*	hypothetical protein	1.05	0.01	2.08
	*BTR42_07470*	hypothetical protein	1.12	0.03	2.17
	*BTR42_07475*	hypothetical protein	1.18	0.00	2.27
	*ssb*	ssDNA-binding protein	1.43	0.03	2.69
	*traG*	TraG protein	1.19	0.02	2.28
UCN 34: Increased gene expression					
function	gene	protein	log2 Ratio	p-value	fold-change
aminoacid metabolism	*ilvC*	ketol-acid reductoisomerase	1.01	0.00	2.01
	*ilvH*	acetolactate synthase (small subunit)	1.04	0.05	2.05
	*GALLO_0983*	putative LrgA protein family	1.07	0.04	2.10
DNA binding	*GALLO_2218*	putative FtsK/SpoIIIE family protein	1.01	0.03	2.01
nucleotide binding	*folC*	putative folyl-polyglutamate synthetase	1.16	0.04	2.23
	*GALLO_0337*	putative dioxygenases related to 2-nitropropane dioxygenase	1.20	0.03	2.29
protease	*GALLO_0591*	putative peptidase	1.58	0.04	3.00
transcriptional regulator	*GALLO_2176*	putative transcriptional regulator; repressor of the trehalose operon	1.07	0.05	2.10
	*GALLO_1670*	putative transcriptional regulator, Cro/CI family	1.20	0.04	2.30
transporter	*GALLO_0120*	putative PTS system, mannose-specific IID component	1.05	0.05	2.07
	*GALLO_2110*	putative permeases	1.06	0.02	2.08
	*GALLO_2083*	putative major facilitator superfamily transport protein	1.12	0.04	2.17
	*GALLO_0891*	putative major facilitator superfamily protein	1.18	0.04	2.26
	*GALLO_1734*	Major Facilitator Superfamily protein	1.23	0.03	2.34
	*GALLO_2083*	putative major facilitator superfamily transport protein	1.25	0.04	2.38
	*nrgA*	ammonium transporter	1.40	0.01	2.64
	*GALLO_1593*	putative MATE family multidrug efflux pumps	1.03	0.04	2.04
tRNA modification	*tgt*	queuine tRNA-ribosyltransferase	1.22	0.05	2.33
unknown function	*GALLO_2204*	putative lipase	1.02	0.05	2.03
	-	hypothetical protein	1.05	0.04	2.08
	*GALLO_0703*	hypothetical protein	1.10	0.04	2.15
	*GALLO_0875*	conserved hypothetical integral membrane protein	1.12	0.05	2.17
	*GALLO_1423*	conserved hypothetical protein	1.14	0.04	2.20
	*GALLO_0720*	conserved hypothetical protein	1.14	0.05	2.21
	*GALLO_1659*	conserved hypothetical secreted protein	1.35	0.03	2.55
	*GALLO_0890*	conserved hypothetical secreted protein	1.36	0.03	2.57

n = 3

### Verification of the microarray analysis of collagen adhesion with relative quantitative real-time PCR

**Fig 5:**
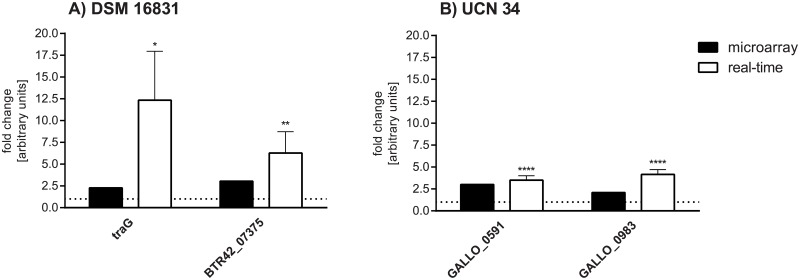
Verification of gene expression changes determined by microarray analysis of bacterial cells adhered to collagen with relative quantitative real-time PCR. The fold change of the gene expression of distinct genes (x-axis) identified by microarray analysis (black) and real-time PCR (white) is represented for DSM 16831 (A) and UCN 34 (B). The dotted line represents the relative mRNA-level in the control which is set by one. Statistical significance between the control mRNA abundances (planktonic; set as one) and the mRNA abundances of collagen-adhered cells are marked with stars (Mann-Whitney *U* test, *: p < 0.05; **: p < 0.005; ****: p < 0.0001; n = 8).

The genes which were found to be regulated by collagen adherence using microarray analysis were mostly specific for the genome of the particular strain of *S*. *gallolyticus* subsp. *gallolyticus*. The phage and transposon genes of strain DSM 16831 have not been found in any other strains of *S*. *gallolyticus* subsp. *gallolyticus* with sequenced genomes. The gene expression of two genes per isolate analyzed was examined by real-time PCR, and results are shown in Fig 5. Genes of interest were only tested in the respective strain and not in the other one, because they were not included in the other genome. The real-time PCR revealed higher changes in gene expression of strain DSM 16831 than the analysis by microarray; the genes examined in this strain coded for transposon proteins. Almost the same regulation in gene expression for strain UCN 34 was observed by relative quantitative real-time PCR and microarray analysis. The genes analyzed in strain UCN 34 code for a putative LrgA protein family protein, which is membrane-associated and a putative peptidase.

## Discussion

This study analyzed the adherence to collagen and biofilm formation of *S*. *gallolyticus* subsp. *gallolyticus* under different conditions. To our knowledge, it is the first time that increased biofilm formation in the presence of lysozyme compared to control has been observed for a pathogen. By contrast, a decrease in biofilm formation was shown following treatment with immobilized and soluble lysozyme in other species, like *S*. *aureus*, *Pseudomonas aeruginosa* and *E*. *coli* [27,42]. Thereby, the strain BAA-2069 generated a biofilm more rapidly by lysozyme treatment compared to other strains. It is well-known that biofilm formation is a survival strategy of bacteria, because they are more resistant against harsh conditions, for example, antibiotics or H_2_O_2_, in this bacteria-embedded community [43,44]. This leads to the hypothesis that biofilm formation while undergoing lysozyme treatment is a defense mechanism of *S*. *gallolyticus* subsp. *gallolyticus* to this bactericidal agent. H_2_O_2_ is also an inducer of increased biofilm formation by *Acinetobacter oleivorans* [3]. In this study, it was determined that H_2_O_2_ is only a slight trigger for biofilm formation in *S*. *gallolyticus* subsp. *gallolyticus* strains UCN 34 and ATCC 43143. This leads to the assumption that lysozyme and H_2_O_2_ function as inducers for biofilm formation of *S*. *gallolyticus* subsp. *gallolyticus* which is of benefit for the pathogen to survive within the host. Contrary results between the analysis with crystal violet, which includes the quantification of all bacteria at the well bottom, and plating assay, which includes the quantification of viable bacteria at the well bottom, could be explained by the observations of Sjomella et al. who revealed that the number of viable bacterial cells decreased when the total number of cells (viable and dead) increased [45].

The initial stage in the pathogenesis of endocarditis is bacterial adherence to an extracellular matrix which is exposed through damaged endothelium [8]. The adhesion ability to collagen types I and IV was studied because type I is abundant in the damaged human heart valve and type IV within epithelial tumors of the colon [15,46]. It was shown that the ability of *S*. *gallolyticus* subsp. *gallolyticus* to adhere to collagen depends highly on the tested strain [20,21]. Boleij et al. postulated that the collagen-binding ability is the key virulence feature of *S*. *gallolyticus* subsp. *gallolyticus* [15]. We have shown that human isolates have significantly higher adhesion ability to collagen than isolates from animals. A reason could be the presence of genes like *pilB* of Pil1 as Danne et al postulated [23]. Comparing adhesion ability and presence of relevant genes, no correlation could be found [20]. For example, the isolate 000718/98 has high adhesion ability but lacks the pilB gene. This leads to the assumption that collagen adherence depends on more proteins than pil1 and is a multifactorial process. Therefore, microarray analysis of collagen adhesion was performed.

Contrary to the biofilm formation triggered by lysozyme on polystyrene, only strain BAA-2069 showed higher biofilm formation at collagen type I by lysozyme treatment compared to control without lysozyme. It is worth considering the consequences of lysozyme as an inducer for biofilm formation *in vivo*. Collagen is exposed by an infection of the endocardium due to damaged endothelium and activated macrophages secrete lysozyme [8,24,26]. Therefore, strain BAA-2069 could form a biofilm more rapidly, thus the strain is better protected against the defense mechanisms of the host.

This is the first study analyzing lysozyme resistance in association with biofilm formation of *S*. *gallolyticus* subsp. *gallolyticus* using a full genome microarray. Lysozyme resistance in streptococci was found to be caused by modifications of peptidoglycan [29]. Genes which are associated with these modifications have not been found in *S*. *gallolyticus* subsp. *gallolyticus* by BLAST analysis. The transcriptome analysis in this study revealed that resistance to lysozyme is probably due to the expression of genes of the *dlt* operon (*dltABCD*). The products of this operon are responsible for the D-alanylation of teichoic acids, which leads to resistance of the cationic antimicrobial peptide activity of lysozyme [28,47].

Furthermore, this study revealed that the expression of transcription- and translation-associated genes is particularly increased compared to control. This was also observed in a growth phase-dependent analysis in *Streptococcus pyogenes* [48]. The treatment with lysozyme could impair the growth of *S*. *gallolyticus* subsp. *gallolyticus* by lysing some bacterial cells. This would lead to differences in the growth between the bacterial cells in BHI and BHI supplemented with lysozyme. Genes of stress response, such as the microcin immunity protein (*mccF*) and the competence induced protein A (*cinA*), also showed higher expression following lysozyme treatment. MccF provides resistance to heptapeptide-nucleotide microcin C, a potent inhibitor of enteric bacterial growth. CinA is a competence protein which is important for natural transformation and for adaptation to the rumen [49,50]. It is known that CinA and exogenous DNA-binding protein (ComEA) are both involved in natural competence [51] and CinA expression is regulated by ComX, the expression of which is regulated in turn by ComEA. Therefore, it is remarkable that expression of *comEA* is decreased whereas the expression of *cinA* is increased by lysozyme treatment.

Additionally, the abundance of transcripts of genes encoding DNA repair proteins are increased. This indicates stress response, but 6-O-methylguanine DNA methyltransferase (Ogt) and DNA repair protein RecO are also relevant for resistance and virulence [52,53]. Interestingly, genes which are involved in resistance to reactive oxygen species are expressed higher in both strains of *S*. *gallolyticus* subsp. *gallolyticus* in the presence of lysozyme, this includes the NADH oxidase NaoX, and the alkyl hydroperoxide reductase (Ahp) C and F [54]. Furthermore, *ahpC* is expressed differently in exponential and stationary growth and *ahpC* knock-out mutants of other bacteria show an increase in biofilm formation [55,56].

*S*. *gallolyticus* subsp. *gallolyticus* strain UCN 34 showed increased expression of transporter genes in the presence of lysozyme, including iron transporter genes. Gene expression of this type of transporter was also induced in the presence of low H_2_O_2_ in *Enterococcus faecalis* and by heat shock of *Streptococcus thermophiles* [57,58]. These results indicate that stress mechanisms which are involved in survival in the presence of reactive oxygen species must also be relevant in resistance to lysozyme.

The adhesion ability to collagen type I and IV was strain-dependent. The transcriptome analysis with full genome microarrays revealed differences in gene expression between the human isolate UCN 34 and the isolate DSM 16831 from koala feces following adhesion to collagen type I. The isolate UCN 34 changed the metabolism to the uptake of nutrients by transporters. The change of nutrients and differences in growth phase between planktonic and adhered bacterial cells provides evidence that the collagen-adhered bacteria analyzed stay within a biofilm [59]. Although *S*. *gallolyticus* subsp. *gallolyticus* builds a biofilm at collagen, the gene expression of the competence protein ComD, which is relevant in streptococcal quorum sensing in biofilms, decreased after adhesion to collagen [51]. The gene expression of two regions in the genome of *S*. *gallolyticus* subsp. *gallolyticus* strain DSM 16831 was increased after adhesion to collagen. One region consists of transposon genes, and has similarities to ICE *Sp*1116 of *Streptococcus pyogenes* [60,61]. The phage genes whose expression was upregulated through collagen adhesion (the second region) have similarities to the streptococcal bacteriophage P9 [38]. Additionally, this is the only complete phage region in strain DSM 16831, but no lysis of bacterial cells was observed in a plaque test. The same insensitivity to this bacteriophage was shown for *Streptococcus zooepidemicus* [62]. The higher abundance of transcripts of both regions leads to the assumption that strain DSM 16831 exchanges DNA when adhered to collagen type I. This was also shown for *Pseudomonas aeruginosa* [63]. This “snapshot” of the transcriptome of strains UCN 34 and DSM 16831 adhered to collagen is consistent with the last phase of general streptococcal adhesion, colonization and biofilm formation described by Nobbs et al. [59].

In conclusion, gene expression of the *dlt* operon could lead to resistance to lysozyme, and lysozyme triggers biofilm formation of *S*. *gallolyticus* subsp. *gallolyticus*, which could be due to the expression of immunity genes, such as *cinA* and *mccF*. Adhesion ability to collagen types I and IV depends on the origin of the respective strain, and collagen adhesion leads to changes in nutrient uptake and DNA exchange.

## Supporting information

S1 File (DOCX)

## Abbreviations

cfu: colony forming units
H_2_O_2_: hydrogen peroxide
IE: infective endocarditis
