# Supplementary material for: Biofilm formation and transcriptome analysis of Streptococcus gallolyticus subsp. gallolyticus in response to lysozyme
Source: PLoS One. 2018 Jan 26;13(1):e0191705. doi: 10.1371/journal.pone.0191705 (PMC5786311; doi:10.1371/journal.pone.0191705)
Supplement: S1 File — (DOCX) [file pone.0191705.s001.docx]

**Supporting Information – S1 File**

Biofilm formation and transcriptome analysis of *Streptococcus gallolyticus* subsp. *gallolyticus* in response to lysozyme

Imke Grimm, Jessika Dumke, Jens Dreier, Cornelius Knabbe and Tanja Vollmer^*^

Institut für Laboratoriums- und Transfusionsmedizin, Herz- und Diabeteszentrum Nordrhein-Westfalen, Universitätsklinikum der Ruhr-Universität Bochum, Bad Oeynhausen, Germany


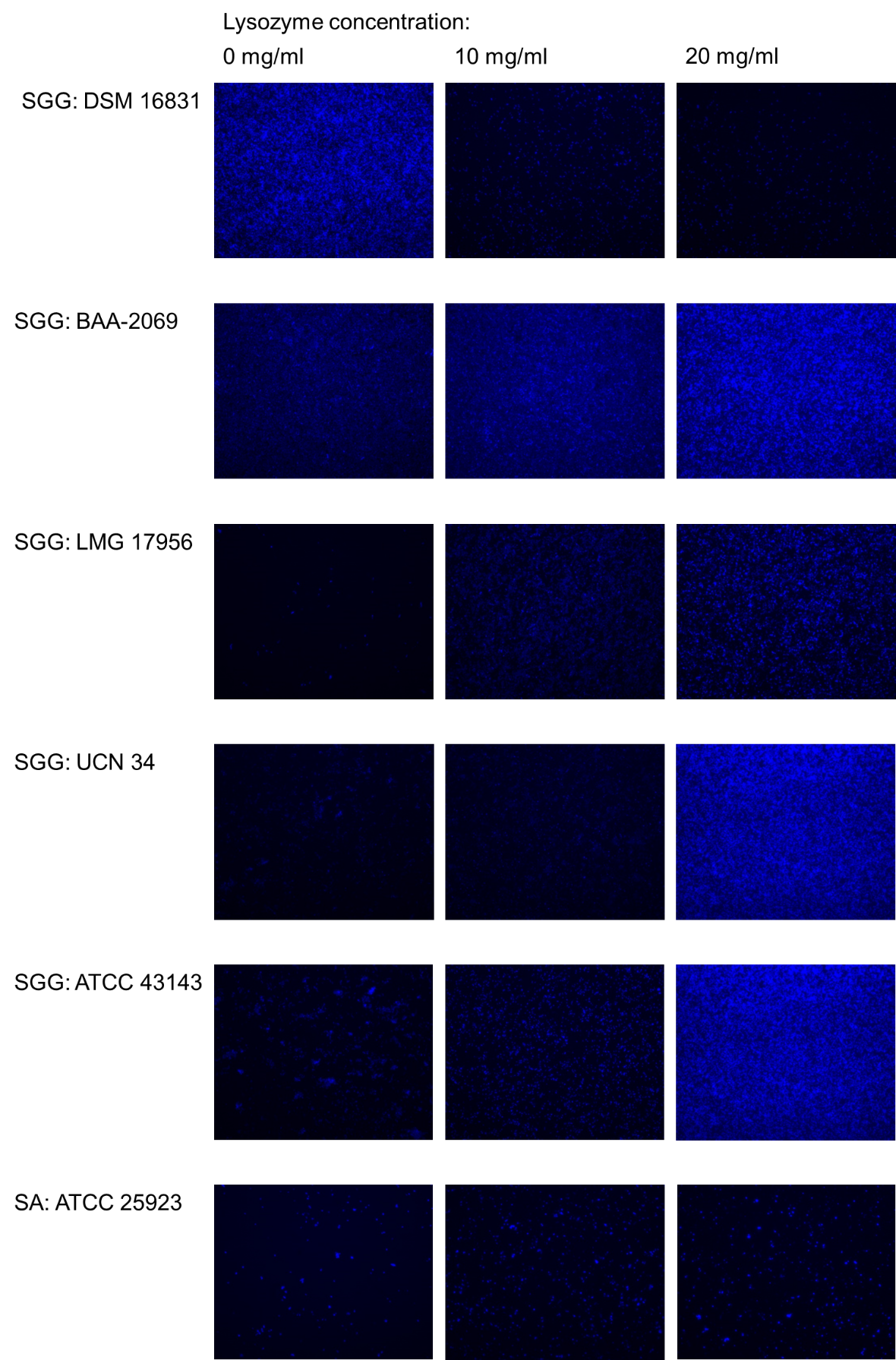


Figure A: **Microscopic analysis of bacterial cells adhered to polystyrene after lysozyme treatment (10 and 20 mg/ml)**

Polystyrene adhered bacterial cells are labeled with DAPI after 16 h of incubation.

Figure B: **Adhesion of *S. gallolyticus* subsp. *gallolyticus* to polystyrene in presence of H_2_O_2_**

Biofilm formation on polystyrene was detected with crystal violet and absorption was determined photometrically after 5 h. Compared is the biofilm formation in BHI without H_2_O_2_ to H_2_O_2_ supplemented medium. Statistical significance between the different time points of a strain is marked with stars (Mann-Whitney U test, *: p < 0.05; **: p < 0.005; n = 3). The standard deviation is marked with error bars. SGG = *Streptococcus gallolyticus* subsp. *gallolyticus*; SA = *Staphylococcus aureus*

Figure C: **Adhesion of *S. gallolyticus* subsp. *gallolyticus* to collagen type I and IV**

Adhesion to collagen type I (A) and IV (B) was detected with crystal violet and absorption was determined photometrically after 2 h. The strains DSM 16831, BAA-2069, LMG 17956, UCN 34 and ATCC43143, which were used in the lysozyme biofilm formation test, are underlined (grey bars). Strains are divided into high adhesion ability (> 1), medium adhesion ability (0.1 – 1) and low or no adhesion ability (< 0.1). Mean with standard error is shown.

Figure D: **Adhesion of *S. gallolyticus* subsp. *gallolyticus* to collagen type I in presence of lysozyme**

Adhesion to collagen type I (A) and type IV (B) was detected with crystal violet and absorption was determined photometrically after 5 h of incubation. Statistical significance is compared between the different time points of a strain is marked with stars (Mann-Whitney U test, *: p < 0.05; **: p < 0.005; n = 3). The standard deviation is marked with error bars. SGG = *Streptococcus gallolyticus* subsp. *gallolyticus*; SA = *Staphylococcus aureus*

Table A: **List of bacterial strains used in this study with source**

| Species | Strain | Origin | Source |
| --- | --- | --- | --- |
| *Streptococcus gallolyticus* subsp. *gallolyticus* | DSM 16831 | coala faeces | DSMZ |
| *Streptococcus gallolyticus* subsp. *gallolyticus* | BAA-2069 | human, IE patient | HDZ |
| *Streptococcus gallolyticus* subsp. *gallolyticus* | LMG 17956 | calf | LMG |
| *Streptococcus gallolyticus* subsp. *gallolyticus* | UCN 34 | human, IE patient | Calvados, France ^29^ |
| *Streptococcus gallolyticus* subsp. *gallolyticus* | ATCC 43143 | human, blood | ATCC |
| *Staphylococcus aureus* | ATCC 25923 | human, clinical isolate | ATCC |
| *Streptococcus gallolyticus* subsp. *gallolyticus* | DSM 13808 | Sapropel | DSMZ |
| *Streptococcus gallolyticus* subsp. *gallolyticus* | Isolate 12932/01 | Human, IE patient | HDZ |
| *Streptococcus gallolyticus* subsp. *gallolyticus* | Isolate 000718/98 | Human, IE patient | HDZ |
| *Streptococcus gallolyticus* subsp. *gallolyticus* | Isolate 010288/01 | Human, IE patient | HDZ |
| *Streptococcus gallolyticus* subsp. *gallolyticus* | Isolate 003080/00 | Human, IE patient | HDZ |
| *Streptococcus gallolyticus* subsp. *gallolyticus* | Isolate 010672/01 | Human, IE patient | HDZ |
| *Streptococcus gallolyticus* subsp. *gallolyticus* | Isolate 006718/00 | Human, IE patient | HDZ |
| *Streptococcus gallolyticus* subsp. *gallolyticus* | Isolate 007849/02 | Human, IE patient | HDZ |
| *Streptococcus gallolyticus* subsp. *gallolyticus* | Isolate 021702/06 | Human, IE patient | HDZ |
| *Streptococcus gallolyticus* subsp. *gallolyticus* | ATCC 49475 | Human, clinical isolate | ATCC |
| *Streptococcus gallolyticus* subsp. *gallolyticus* | ATCC 49147 | Human, clinical isolate | ATCC |
| *Streptococcus gallolyticus* subsp. *gallolyticus* | 0134287/1 | Human, IE patient | Unk. |
| *Streptococcus gallolyticus* subsp. *gallolyticus* | ATCC 9809 | Unk. | ATCC |
| *Streptococcus gallolyticus* subsp. *gallolyticus* | Isolate 005950/03 | Human, IE patient | HDZ |
| *Streptococcus gallolyticus* subsp. *gallolyticus* | K6236/35_MS | Human, IE patient | Unk. |
| *Streptococcus gallolyticus* subsp. *gallolyticus* | 05WDK43740002 | Bovine | Unk. |
| *Streptococcus gallolyticus* subsp. *gallolyticus* | AC 6860 | Human, clinical isolate | RWTH |
| *Streptococcus gallolyticus* subsp. *gallolyticus* | AC 1016 | Human, clinical isolate | RWTH |
| *Streptococcus gallolyticus* subsp. *gallolyticus* | AC 1135 | Human, clinical isolate | RWTH |
| *Streptococcus gallolyticus* subsp. *gallolyticus* | AC 1181 | Human, clinical isolate | RWTH |
| *Streptococcus gallolyticus* subsp. *gallolyticus* | AC 1242 | Human, clinical isolate | RWTH |
| *Streptococcus gallolyticus* subsp. *gallolyticus* | AC 582 | Human, clinical isolate | RWTH |
| *Streptococcus gallolyticus* subsp. *gallolyticus* | AC 7070 | Human, clinical isolate | RWTH |
| *Streptococcus gallolyticus* subsp. *gallolyticus* | AC 6827 | Human, clinical isolate | RWTH |
| *Streptococcus gallolyticus* subsp. *gallolyticus* | LMG 14621 | Pigeon, abscess | LMG |
| *Streptococcus gallolyticus* subsp. *gallolyticus* | LMG 14622 | Pigeon | LMG |
| *Streptococcus gallolyticus* subsp. *gallolyticus* | LMG 14623 | Pigeon, liver | LMG |
| *Streptococcus gallolyticus* subsp. *gallolyticus* | LMG 14634 | Bovine, intestine | LMG |
| *Streptococcus gallolyticus* subsp. *gallolyticus* | LMG 14821 | Pigeon | LMG |
| *Streptococcus gallolyticus* subsp. *gallolyticus* | LMG 14855 | Horse, intestine | LMG |
| *Streptococcus gallolyticus* subsp. *gallolyticus* | LMG 14856 | Horse, intestine | LMG |
| *Streptococcus gallolyticus* subsp. *gallolyticus* | LMG 14870 | Bovine, intestine | LMG |
| *Streptococcus gallolyticus* subsp. *gallolyticus* | LMG 14876 | Bovine, tonsil | LMG |
| *Streptococcus gallolyticus* subsp. *gallolyticus* | LMG 14878 | Pigeon, lung | LMG |
| *Streptococcus gallolyticus* subsp. *gallolyticus* | LMG 15572 | Wild goat, rumen | LMG |
| *Streptococcus gallolyticus* subsp. *gallolyticus* | LMG 15573 | Wild goat, rumen | LMG |
| *Streptococcus gallolyticus* subsp. *gallolyticus* | LMG 16005 | Bovine, intestine | LMG |
| *Streptococcus gallolyticus* subsp. *gallolyticus* | LMG 22782 | Dog | LMG |
| *Streptococcus gallolyticus* subsp. *gallolyticus* | LMG 14625 | Human, blood culture | LMG |
| *Streptococcus gallolyticus* subsp. *gallolyticus* | LMG 14631 | Human, blood culture | LMG |
| *Streptococcus gallolyticus* subsp. *gallolyticus* | LMG 14632 | Human, blood culture | LMG |
| *Streptococcus gallolyticus* subsp. *gallolyticus* | LMG 14823 | Pigeon | LMG |
| *Streptococcus gallolyticus* subsp. *gallolyticus* | LMG 14879 | Pigeon, lung | LMG |
| *Streptococcus gallolyticus* subsp. *gallolyticus* | LMG 15049 | Bovine, mastitis | LMG |
| *Streptococcus gallolyticus* subsp. *gallolyticus* | LMG 15053 | Bovine, mastitis | LMG |
| *Streptococcus gallolyticus* subsp. *gallolyticus* | LMG 15063 | Bovine, mastitis | LMG |
| *Streptococcus gallolyticus* subsp. *gallolyticus* | LMG 17082 | Bovine, tonsil | LMG |
| *Streptococcus gallolyticus* subsp. *gallolyticus* | LMG 17091 | Bovine, tonsil | LMG |
| *Streptococcus gallolyticus* subsp. *gallolyticus* | Sp 001 | Human, blood culture | IRYCIS |
| *Streptococcus gallolyticus* subsp. *gallolyticus* | Sp 006 | Human, blood culture | IRYCIS |
| *Streptococcus gallolyticus* subsp. *gallolyticus* | Sp 012 | Human, blood culture | IRYCIS |
| *Streptococcus gallolyticus* subsp. *gallolyticus* | Sp 014 | Human, blood culture | IRYCIS |
| *Streptococcus gallolyticus* subsp. *gallolyticus* | Sp 015 | Human, blood culture | IRYCIS |
| *Streptococcus gallolyticus* subsp. *gallolyticus* | Sp 020 | Human, blood culture | IRYCIS |
| *Streptococcus gallolyticus* subsp. *gallolyticus* | Sp 023 | Human, blood culture | IRYCIS |
| *Streptococcus gallolyticus* subsp. *gallolyticus* | Sp 029 | Human, blood culture | IRYCIS |
| *Streptococcus gallolyticus* subsp. *gallolyticus* | Sp 030 | Human, blood culture | IRYCIS |
| *Streptococcus gallolyticus* subsp. *gallolyticus* | Sp 036 | Human, blood culture | IRYCIS |
| *Streptococcus gallolyticus* subsp. *gallolyticus* | Sp 042 | Human, blood culture | IRYCIS |
| *Streptococcus gallolyticus* subsp. *gallolyticus* | Sp 043 | Human, blood culture | IRYCIS |
| *Streptococcus gallolyticus* subsp. *gallolyticus* | Sp 050 | Human, blood culture | IRYCIS |
| *Streptococcus gallolyticus* subsp. *gallolyticus* | Sp 052 | Human, blood culture | IRYCIS |
| *Streptococcus gallolyticus* subsp. *gallolyticus* | Sp 053 | Human, blood culture | IRYCIS |
| *Streptococcus gallolyticus* subsp. *gallolyticus* | 904 | Human, IE patient | Korea |
| *Streptococcus gallolyticus* subsp. *gallolyticus* | 9914B3313 | Human, blood culture | Unk. |
| *Streptococcus gallolyticus* subsp. *gallolyticus* | Isolate 52 F2 | Human, feces | HDZ |
| *Streptococcus gallolyticus* subsp. *gallolyticus* | Isolate 52 G9 | Human, feces | HDZ |
| *Streptococcus gallolyticus* subsp. *gallolyticus* | 99208B2062 | Human, blood culture | Unk. |
| *Streptococcus gallolyticus* subsp. *gallolyticus* | 013425/1 | Unk. | Unk. |
| *Escherichia coli* | TG1 | Unk. | Unk. |
| *Lactococcus lactis* | 15976/05 | Unk. | HDZ |

DSMZ: German Collection of Microorganisms and Cell Cultures; LMG: Laboratory of Microbiology, Ghent University; ATCC: American Type Culture Collection; HDZ: Heart and Diabetes Centre NRW; RWTH: RWTH Aachen University; LMG: Laboratory of Microbiology, Ghent University; IRYCIS: “Servicio de Microbiología, Instituto Ramón y Cajal de Investigación Sanitaria, Hospital Universitario Ramón y Cajal”, Madrid; Unk.: Source or origin unknown

Table B: **Oligonucleotides used for relative quantitative real-time PCR analysis**

| *S. gallolyticus* subsp. *gallolyticus* | |
| --- | --- |
| Product name (gene name) | 5′–3′ sequence: Forward  Reverse |
| 16S ribosomal RNA (*16S*) | TTATGACCTGGGCTACAC  CCTACAATCCGAACTGAG |
| 23S ribosomal RNA (*23S*) | CTKCCAAGAAAAGCYTCT  CATTTTGCCDAGTTCCTT |
| alanyl-tRNA synthetase (*alaS*) | GCTGCTACGCTTAAAGTC  GTTGTCCGCAAATGTACG |
| F0F1 ATP synthase subunit A (*atpB*) | GCCGATGAACATTCTCGAAG  TGAGAACCCTGTCCAAAG |
| Competence damage-inducible protein A (*cinA*) | GCTTCGCCCACCTATAAC  GTTGCAGGACCAGATAGC |
| Exogenous DNA-binding protein (*comEA*) | ATGCGCAAAGTGACCTACCTG  CCGCCAAAGTTGCGGTATTAAG |
| D-alanine transfer from Dcp to undecaprenol-phosphate (*dltB*) | AGCTGTGGCAATAGCAAGCTAC  ACGGCGTGCTGAGTGAATTTA |
| D-alanine transfer from undecaprenol-phosphate to the poly(glycerophosphate) chain (*dltD*) | CCACCGAGTTTGCTGTAAG  GTGTTTATGACCGCTACGC |
| Peroxide resistance protein (*dpr*) | TGATGGACGGTCTTAACG  CTTGGAGCATCCAGATTG |
| Iron complex transport system ATP-binding protein (*fhuC*) | CACGCATTCATGCCCTTAAAGC  ATGTGGAAAGCGACCGTATGAG |
| Nitrogen regulatory protein PII (*gnlB*) | TCGTTCTCCTGTCCGAATAC  CGGCGGTTGATGAAATTGTG |
| Microcin immunity protein (*mccF*) | CCACCAATCGTAGCCAGAATAG  AGTCACATTCGCGTACTCAG |
| Multidrug rsistance protein (*norN*) | AAGTCAATTGCCGATGGG  ACCAGCACGCATCATTAG |
| post-translocation molecular chaperone (*prsA1*) | TATCGGTGCTGGTGTAAC  GTGCGCTTGAGAATGATG |
| 50S ribosomal protein L1 (*rplA*) | CGATAGCCATCATGTCTG  GCGTAGAAGAAGCTGTAG |
| Conjugal transfer protein TraG (*traG*) | GCTGCTACGCTTAAAGTC  GTTGTCCGCAAATGTACG |
| *BTR42_07375* | TAGGGTTGGTGTTCGATCAG  GCAACCGCTTCAGGAGTTATTC |
| peptidase (*GALLO_0591*) | AGACTCAAGCTGGCAAGGTAG  TGTTGGTTTGGCTCCTTCC |
| LrgA protein family (*GALLO_0983*) | ACAAAACCGATAACGGCAACA  CCGCCTTAGACACATTGACG |
